# Assessment of the satisfaction with public health insurance programs by patients with chronic diseases in China: a structural equation modeling approach

**DOI:** 10.1186/s12889-021-11947-7

**Published:** 2021-10-19

**Authors:** Jinsong Geng, Xiaowei Chen, Jianwei Shi, Haini Bao, Qian Chen, Hao Yu

**Affiliations:** 1 Medical School of Nantong University, Nantong, 226001 Jiangsu China; 2 Library and Reference Department, The First Affiliated Hospital of Zhejiang University School of Medicine, Zhejiang, 310003 Hangzhou China; 3 School of Public Health, Shanghai Jiaotong University School of Medicine, Shanghai, 200025 China; 4 Department of Ophthalmology, The Affiliated Hospital of Nantong University, Nantong, 226001 Jiangsu China; 5 Department of Population Medicine, Harvard Medical School and Harvard Pilgrim Health Care Institute, Boston, MA 02215 USA

**Keywords:** Chronic disease, Patient satisfaction, Health insurance, Structural equation modeling

## Abstract

**Background:**

China has successfully sustained its universal health insurance coverage over the past decade. Although patient satisfaction has been recognized as an important indicator to measure the performance of insurance programs in China, there is a lack of evidence on how patients with chronic diseases are satisfied with China’s public health insurance programs and whether their satisfaction differs by type of insurance. We aimed to fill the evidence gap.

**Methods:**

We established a hypothetical model that comprised patients’ awareness of insurance policies, the fulfillment of patients’ expectations of insurance benefits, patients’ perceived value of health insurance coverage, patients’ satisfaction with health insurance programs, patients’ complaints, and trust in health insurance programs. We performed a confirmatory factor analysis by using a structural equation modeling (SEM) approach to examine the hypothesized model. A model-testing survey in 10 tertiary hospitals was conducted between June and October 2018, with a valid sample of 922 insured patients with chronic diseases.

**Results:**

The SEM model, with good fit indices, showed that patients’ awareness of health insurance policies, insurance program’s fulfillment of expectations, and patients’ perceived value of insurance coverage, positively predicted patient satisfaction (*P* < 0.01). The fulfillment of patients’ expectations of insurance benefits was the major predictor of satisfaction with health insurance (coefficient = 0.593, *P* < 0.001), while the patients’ perceived value of insurance coverage had the largest impact on their trust in health insurance (coefficient = 0.409, *P* < 0.01). Compared to patients with Urban-Rural Resident Basic Medical Insurance, Urban Employee Basic Medical Insurance enrollees had a higher degree of satisfaction with insurance on average (*P* < 0.01). Despite differences in the degree of satisfaction, the main findings from the SEM were also proved by the multi-group analysis.

**Conclusions:**

Our findings highlight the importance of incorporating patients’ perceived value as part of the ongoing efforts to increase satisfaction with health insurance by patients, especially those who have chronic diseases. Policymakers are also suggested to formulate evidence-informed reimbursement policies that meet patients’ expectations.

**Supplementary Information:**

The online version contains supplementary material available at 10.1186/s12889-021-11947-7.

## Background

Chronic diseases pose a daunting challenge to the healthcare system in China. Almost half of the Chinese adults aged 35–75 years have hypertension, but less than one-third of them are being treated [1]. China was also projected to have 116 million diabetes patients in 2019 [2]. Meanwhile, chronic diseases are associated with high costs. It was estimated that the total economic costs of chronic diseases in China would be US$16 trillion (measured in 2010 US Dollars) for the period of 2010–2030 [3].

In response to the rising challenges, China’s central government initiated a nationwide healthcare reform in 2009, aiming to develop a universal basic health insurance system and offer safe, effective, convenient, and affordable healthcare services for all its populations, especially those who have chronic diseases. By the end of 2011, China successfully achieved universal health insurance coverage (UHC) through three major public health insurance programs, including Urban Employee Basic Medical Insurance (UEBMI), New Rural Cooperative Medical Scheme (NRCMS), and Urban Resident Basic Medical Insurance (URBMI) [4]. Since then, Chinese governments have taken further reform measures with priorities on broadening the scope of insurance benefit coverage, increasing fiscal subsidies, financing levels, and reimbursement rates, as well as improving provider payment mechanisms [5]. As a key step of further reform, NRCMS and URBMI merged to form the Urban-Rural Resident Basic Medical Insurance (URRBMI) in 2016 to improve administrative efficiency and reduce inequality in insurance benefits. In spite of the reform, disparities in insurance benefits and patients’ utilization of healthcare services still exist [6, 7]. For example, one recent study highlighted gaps in the quality of care, control of chronic diseases, efficiency in delivery, control of health expenditures, and public satisfaction [8].

While the published studies have focused on patient satisfaction with healthcare, there is a lack of studies of satisfaction among chronic disease patients with public health insurance programs in China [9]. It is important to examine patient satisfaction with health insurance programs for several reasons. First, UHC policies should be tailored to patients’ needs and expectations as recommended by World Health Organization [10]. Second, assessment of patient satisfaction and involving patients in health insurance decision-making is an integral part of patient-centered care. Third, as part of its latest policies to strengthen public health insurance programs, China’s central government indicated that one of its primary goals is to improve satisfaction with public health insurance programs by its population [11].

This study presents findings from a 2018 survey of patient satisfaction with China’s public health insurance programs, which can be used as baseline information for future research on whether China’s new policies achieve the goal of improving patient satisfaction with public insurance. Specifically, we took a structural equation modeling (SEM) approach, a series of statistical methods that allow for analyzing complex relationships between one or more independent and dependent variables. Due to its methodological advantages, SEM has been widely used in healthcare services and outcomes research [12–14]. Since insurance coverage is particularly important for patients with chronic diseases [15], our study added new information to the literature by using SEM to examine the satisfaction with China’s public health insurance programs by patients with chronic diseases, whose ongoing healthcare needs are associated with high costs and impose a heavy burden on the insurance programs.

## Methods

### Research framework

We provided a brief review of the literature below before presenting our theoretical framework. Several studies have applied the SEM approach to assess residents’ satisfaction with health insurance in China [16–22]. However, none of the studies focused on patients with chronic diseases. While the participants varied substantially across the studies, such as elementary students, rural and urban residents [18, 20, 21]. Many of the participants had no experience of getting reimbursement from insurance programs, and consequently what they reported in the studies were not their actual satisfaction with insurance programs, but their expectations of the programs. In addition, the findings were inconsistent across the published studies. For instance, contrary to theoretical predictions, one study found that the expanded scope of insurance coverage had a negative impact on patient satisfaction [16], while the researchers did not offer any convincing explanations.

**Fig. 1:**
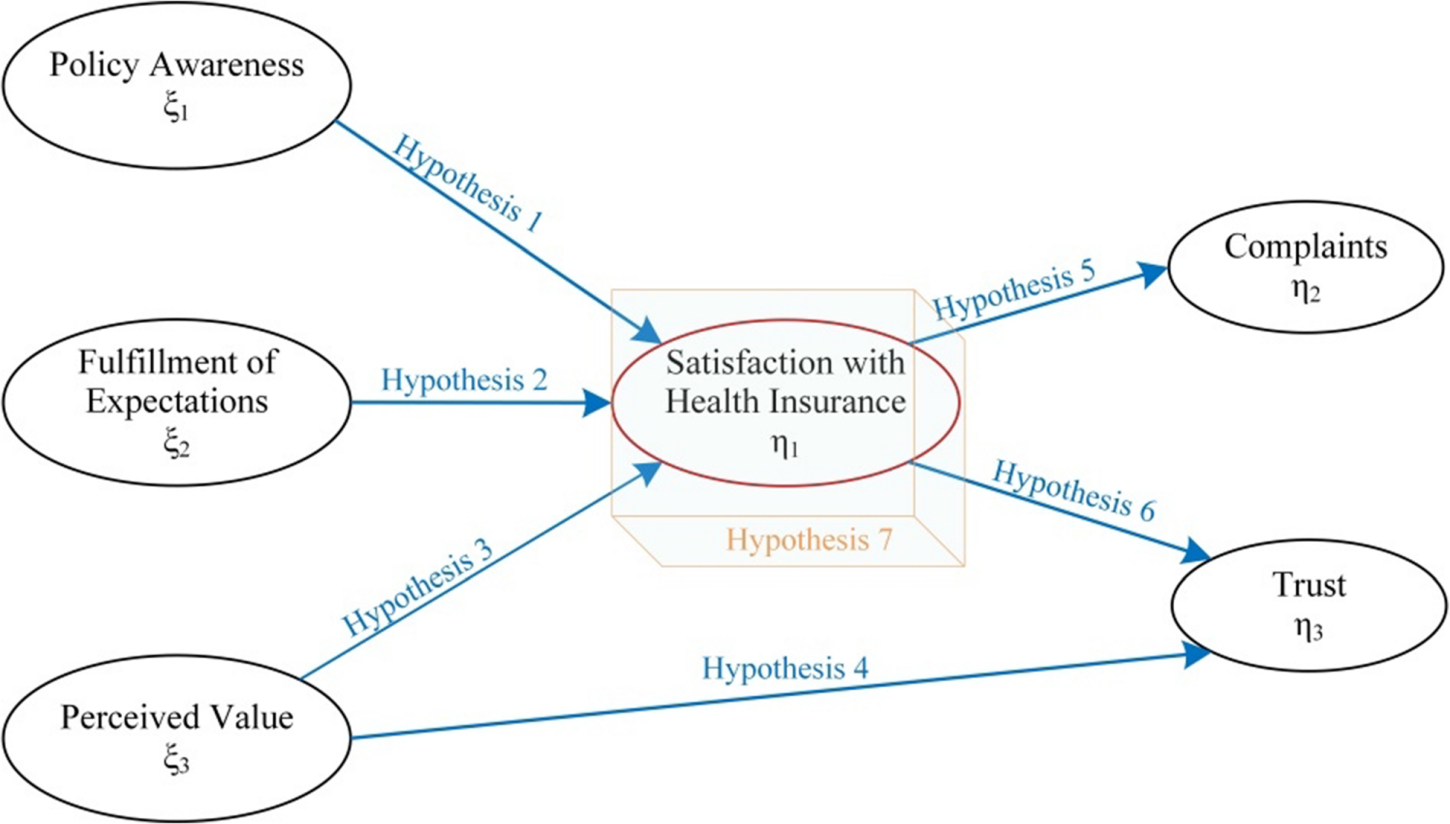
Hypothesized model of patient satisfaction with public health insurance programs. ξ: exogenous variables (variables that are not affected by other variables in the model); η: endogenous variables (variables that are determined by one or more variables in the model). Endogenous variables act as the dependent variable and exogenous variables are equivalent to the independent variables

Drawn from the literature review and the established customer satisfaction theories, we developed a conceptual model (Fig. 1). Previous research found that the degree of awareness of health insurance policies was a factor associated with the satisfaction of healthcare services [23, 24] and that enrollees’ health insurance knowledge and awareness of their premium contributions had a major effect on the satisfaction of insurance programs [25]. Therefore, our first hypothesis in the model is as follows:

*Hypothesis 1: Patients’ awareness of health insurance policies has a direct positive effect on their satisfaction with the health insurance schemes.*

A prior study revealed a significantly positive correlation between meeting patients’ expectations of healthcare at the time receiving healthcare and patients’ satisfaction with the overall experiences of medical service [26]. Prior studies also found that the fulfillment of patients’ expectations of healthcare was associated with greater improvement in patient-reported outcomes and patient satisfaction [27–29]. The unfulfilled expectations, on the contrary, might lead to ongoing concerns and disappointments among patients [30]. Accordingly, we proposed the second hypothesis below:

*Hypothesis 2: Fulfilling patients’ expectations of reimbursement has a direct positive impact on their satisfaction with health insurance.*

The perceived value had significant effects on customers’ satisfaction with the services they received [31, 32]. Specifically, the perceived value of healthcare services has been shown to be an important predictor of satisfaction with the services [33]. Moreover, health insurance enrollees were affected by their perceived value of insurance programs in reaching their loyalty towards the reimbursement schemes [34]. Thus, we propose Hypothesis 3 and 4:

*Hypothesis 3: The patients’ perceived value of health insurance has a direct positive impact on their satisfaction with health insurance.*

*Hypothesis 4: The patients’ perceived value of health insurance has a direct positive effect on their trust in health insurance.*

Increased customer satisfaction resulted in a decrease in their complaints [35]. A previous study also indicated that a decrease in patient satisfaction scores was correlated with increased rates of unsolicited complaints [36]. As a result, patients’ satisfaction with health insurance is likely to have a negative association with their complaints with insurance programs. Prior research also indicated that patients’ satisfaction with the healthcare services had a positive impact on their trust in hospitals [37]. Similarly, we proposed that satisfied insurance enrollees tend to trust their insurance programs. Accordingly, the following hypotheses were formulated.

*Hypothesis 5: Patients’ satisfaction with health insurance has a direct negative effect on their complaints about health insurance.*

*Hypothesis 6: Patients’ satisfaction with health insurance has a direct positive effect on their trust in health insurance.*

We hypothesized earlier that three factors—patients’ health insurance policy awareness, the fulfillment of patients’ expectations of insurance benefits, and the patients’ perceived value of insurance coverage—predicted patients’ satisfaction with health insurance programs. We also proposed that patients with higher levels of satisfaction with their insurance were more likely to trust and less likely to complain about insurance programs. Therefore, we held the view that the indirect effects of the exogenous variables (i.e., the first three factors) on the outcome variables (i.e., trust and complaints) would be significant. Consequently, we proposed the following hypothesis:

*Hypothesis 7: Patients’ satisfaction with health insurance can mediate the effects of the exogenous variables (policy awareness, the fulfillment of expectations, and perceived value) on the final outcome variables (complaints and trust).*

### Measures

#### Patients’ awareness of insurance policies

One of the potential predictors of the enrollees’ satisfaction with health insurance was the awareness of insurance policies such as premium and compensation [38]. Since 2018, China’s public health insurance programs have been regulated by the National Healthcare Security Administration (NHSA), which has decided on policies and has provided guidelines about insurance premiums and reimbursement at the provincial and regional levels, directly impacting patients’ affordability and accessibility to medical technologies and healthcare services [39]. The NHSA not only assumes administrative responsibility for China’s public health insurance programs but also integrates previously separated purchasing powers, including medical care pricing, procurement, and provider payments [40]. Understanding the policies and guidelines issued by the NHSA and its branches at the provincial level is a huge challenge for patients since the policies and guidelines included many specific features by type of insurance programs, such as the catalog of health technologies covered by an insurance program, the ceiling of insurance reimbursement, the deductibles and copayment ratios by type of services, and the reimbursement procedures, which may vary across provinces and cities. Patients’ awareness of these policy components has therefore been considered as measurement variables in our model.

#### The fulfillment of patients’ expectations of insurance benefits

A well-functioning health system responds in a balanced way to a population’s needs and expectations [41]. It is important for an insurance program to fulfill patients’ expectations of insurance benefits [42] since the fulfillment is clearly linked with patient satisfaction [43]. Because the scope of healthcare services coved by an insurance program, the reimbursement and copayment ratios, as well as the reimbursement amount, have been key features of insurance benefits [44, 45], we took such attributes into consideration. In our survey, the scope of covered healthcare services represented the catalog of health technologies issued by the NHSA and its branch in Jiangsu province, the reimbursement ratio referred to the fixed proportion paid by an insurance program for the covered healthcare services, and the reimbursement amount was the payments made by the health insurance program to patients.

#### Patients’ perceived value of health insurance coverage

Patients preferred healthcare services that they perceived as valuable to their overall health needs [46, 47]. For insurance coverage to improve health, insurance programs must improve patients’ care, not just change how the care is paid for [15]. High-value healthcare services are those whose clinical effectiveness is well established, and whose health benefits are judged to be proportional to their cost [48]. However, it is very hard for patients to comprehend the concept of the cost-effectiveness of medical technologies. As shown in patient-centered surveys [49–51], effectiveness and cost were often treated as independent attributes of medical technologies. Consequently, patients’ perceived clinical and economic values of health insurance were considered in our study.

#### Patients’ satisfaction with health insurance programs

Patients are key stakeholders and beneficiaries of health insurance programs. Their views are important for shaping health insurance policies, providing feedback on the quality and responsiveness of insurance programs, and bringing transparency and accountability to the insurance policy-making process. To measure patients’ satisfaction with health insurance, our study included important latent variables, such as the scope of healthcare services covered by an insurance program, reimbursement rates, reimbursement amount by setting (inpatient and outpatient services), and the efficiency of the reimbursement procedure, all of which were selected on the basis of prior study findings [38, 52].

#### Patients’ complaints about health insurance

Patients’ complaints often highlight problems that need to be addressed in the healthcare system [53, 54]. Patients are sensitive to and able to identify a range of problems in the system, some of which are not recognized by conventional methods of health surveillance (e.g., retrospective case reports, incident reporting systems) [55]. Patients’ complaints (sometimes referred to as suggestions, feedback, or grievances) can also offer valuable insights into health insurance by highlighting problems that policymakers may not be aware of. To capture patient sentiment and experience with health insurance programs, we included two variables with one asking about whether a patient had any complaints about his/her insurance program, and the other about whether a patient tried to find solutions to his/her complaints by calling or writing to the NHSA and its branches or other agencies (e.g., Health Commissions at the national, provincial and local levels).

#### Patients’ trust in health insurance programs

Patients’ trust in health insurance schemes is central to policymakers and can be used as a potential marker for how patients evaluate the performance of health insurance. In addition, patients’ willingness to pay for and willingness to join a health insurance plan could reflect the degree of their trust in the insurance schemes [56, 57]. Therefore, we measured patients’ trust in health insurance by using variables about their confidence in the reimbursed medical technologies, their willingness to remain enrolled in health insurance schemes, and their confidence in the sustainability of their insurance schemes.

### Sampling

To test the hypotheses empirically, we performed a survey. The questionnaire consisted of two parts. The first part was about patient socio-demographic characteristics, such as gender, age, education, employment, type of insurance, family monthly income, and medical history. The second part had 22 items representing the above six latent variables (Additional file 1: Table S1). All items were measured by a seven-point Likert scale. For example, for the item of satisfaction with the overall insurance reimbursement experience, the variable was coded as: strongly dissatisfied = 1, dissatisfied = 2, somewhat dissatisfied = 3, neither satisfied nor dissatisfied = 4, somewhat satisfied = 5, satisfied = 6, and strongly satisfied = 7.

Before the pilot study, we consulted experts on the content validity of the questionnaire. Then, the pilot study was conducted between April and May 2018 with 90 patients with chronic diseases participated in the survey. Responses from the patients led to a detailed revision of the survey questions. During the pilot, we also tested the reliability of the questionnaire.

### Field study

Our formal survey was conducted in 10 tertiary public hospitals (Additional file 1: Table S2) in Jiangsu Province between June and October 2018. Inclusion criteria were patients 18 years of age or older, participating in a public health insurance program, who had a history of diabetes, chronic cardiovascular disease, chronic cerebrovascular diseases, or chronic respiratory diseases. Patients were recruited consecutively during the study period.

We decided on our sample size by using the following rules. One is that Mitchell suggested that there should be 10 to 20 times as many cases as variables in SEM analysis [58]. An optimal ratio of 20:1 (number of participants to number of parameters to estimate) was recommended for increased confidence in the results [59]. The SEM questionnaires were administered through one-to-one, face-to-face interviews to ensure the validity and completeness of the investigation. Our interviewers consisted of 13 medical students, all of whom were in hospitals doing their internships during the research period.

### Statistical analysis

Since our objective was to conduct a confirmatory factor analysis, a covariance-based SEM was preferred. The data analysis was undertaken by SPSS 23.0 (IBM Corp., Armonk, NY, USA) and AMOS 24.0 (IBM Corp., Armonk, NY, USA). In the first stage, the reliability and validity of the constructed model and measurement items were tested. The internal consistency was measured using Cronbach’s alpha coefficient. The convergent validity was assessed by factor loading, composite reliability (CR), and average variance extracted (AVE). To ensure discriminant validity, we verified whether the square root of AVE was greater than the correlations of the constructs [60].

The second stage was the goodness-of-fit test, i.e., the substantial differences between the covariance structure observed and the covariance structure implied by the anatomical model. Absolute fit indices, incremental fit indices, and Parsimonious fit indices were used as the criteria to assess the goodness-of-fit [61].

In the third stage, mediation and moderation analysis were conducted. The standardized direct effects, indirect effects, and total effects were estimated using the maximum likelihood method for normal distribution parameters. We used 5000 bootstrap samples with 95% bias-corrected confidence intervals to determine the significance of results and subsequently validated the proposed model. The connection strength (path coefficient) reflects the response of the dependent variable to a unit change in an explanatory variable while the other variables in the model are kept constant [62]. A positive coefficient means that a unit increase in the activity measure of one structure contributes to a direct increase in the activity measure of structures to which it projects, in proportion to the size of the coefficient. While a negative coefficient means that an increase in the activity measure in one structure causes a direct, proportionate decrease in the activity measure of structures it projects to.

Finally, the multi-group analysis was performed by type of insurance (UEBMI and URRBMI) to examine if there were differences in factor loadings, regression weights, and factor variance between the two groups. The measurement weights, structural weights, and structural covariances were constrained to be equal across groups.

## Results

### Demographic characteristics of the study patients

A total of 966 patients consented to participate in the SEM survey. Data from 922 patients were complete and used in the analysis. The 44 patients were excluded from the analysis due to non-compliance with the inclusion criteria, incomplete or invalid data. Table 1 presented the demographic characteristics of the included patients. The sample consisted of more males than females (56.62% vs. 43.38%). The mean age of the patients was 62.93 years (standard deviation 13.11, ranging from 19 to 94 years). There were more UEBMI enrollees than URRBMI enrollees (55.97% vs. 44.03%).

**Table 1:** Demographic characteristics of the study patients (*n* = 922)

Characteristics	n (%)
Gender	
Male	522 (56.62)
Female	400 (43.38)
Age groups	
≤ 34	27 (2.93)
35 ~ 49	101 (10.95)
50 ~ 64	347 (37.64)
65 ~ 79	354 (38.39)
≥ 80	93 (10.09)
Education	
Unschooled	110 (11.93)
Elementary school	249 (27.01)
Middle school	302 (32.75)
High school	149 (16.16)
Junior college or above	112 (12.15)
Employment	
Farmer	198 (21.48)
Urban employee	197 (21.37)
Retiree	348 (37.74)
Freelancer	140 (15.18)
Unemployed	39 (4.23)
Type of insurance	
UEBMI	516 (55.97)
URRBMI	406 (44.03)
Family monthly income (CNY) ^a^	
< 2000	179 (19.41)
2001 ~ 4000	246 (26.68)
4001 ~ 6000	217 (23.54)
6001 ~ 8000	103 (11.17)
8001 ~ 10,000	70 (7.59)
> 10,000	107 (11.61)

^a^The average exchange rate between US Dollars and the Chinese Yuan (CNY) in 2018 was 1: 6.56

### Reliability and validity analyses

The Cronbach’s α ranged from 0.826 to 0.941 for the constructs (Additional file 1: Table S3), which supported the internal consistency of the measurement items. The scales of CR showed a high degree of composite reliability, which was essential for establishing structural relationships between the indicators and the latent variables. The AVE value was higher than the threshold of 0.5, which offered evidence of convergent validity. Factor loading values ranged from 0.603 to 0.947, indicating the construct validity of the measurement scales. The square root of AVE was greater than the correlation involving the constructs (Additional file 1: Table S4), confirming the discriminant validity.

Generally, patients were not well aware of their insurance policies (mean value of measurement ranging from 3.079 to 3.860) and had few complaints about their insurance programs (3.017 to 3.222). Patients expressed a moderate level of their insurance programs’ fulfillment of their expectations of health insurance benefits (3.992 to 4.073) and a moderate level of satisfaction with their insurance programs (4.039 to 4.393). Measurement variables with the highest score were patients’ perceived value of insurance coverage (5.547 to 5.700) and trust in insurance (5.194 to 5.680).

### Goodness-of-fit analysis

Since the hypothesized measurement model provided an adequate representation of the data, reflecting a good fit, no further modification of the model was needed (Additional file 1: Table S5). There was sufficient normality on multivariate tests, with the absolute value of skewness was less than 1.0, and the kurtosis value was less than 1.5. We also found a Kaiser-Meyer-Olkin value of 0.880 and a *P*-value less than 0.001 in Bartlett’s test of sphericity, indicating the suitability of data for SEM.

### Confirmatory factor analysis of the hypothesized structural model

**Fig. 2:**
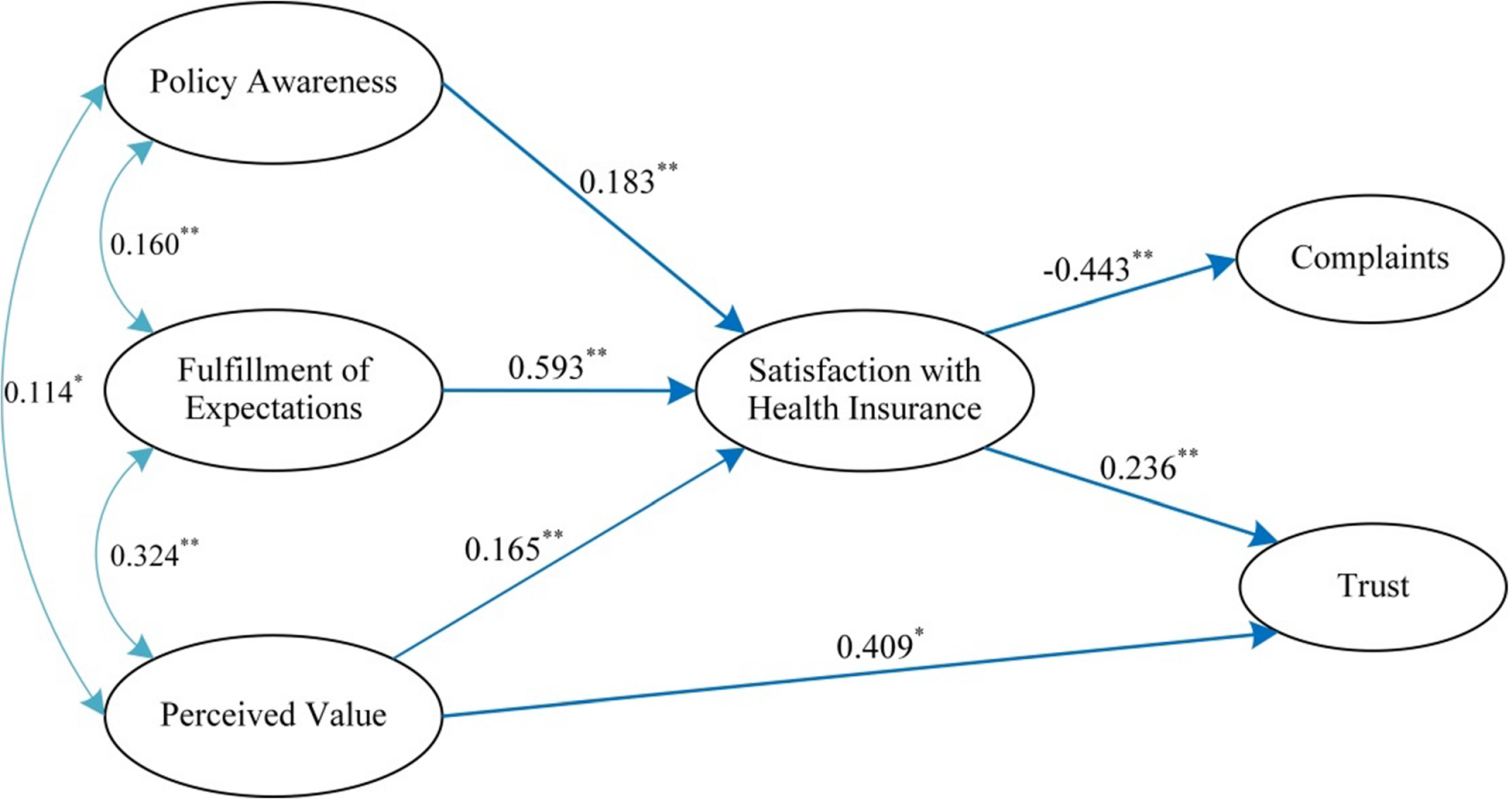
Structural path estimates of the hypothesized model**.** Single arrows represent the standardized parameter estimates of the direct effects, and multi-headed arrows represent the standardized correlations. To simplify the presentation of the model, the manifest variables have been omitted.*P < 0.01, **P < 0.001

The standardized path coefficients for all six latent variables were statistically significant (*P* < 0.01) (Fig. 2). Patients’ awareness of health insurance policies and their insurance programs’ fulfillment of their expectations of insurance benefits positively influenced their satisfaction with insurance (*P* < 0.001) (Hypothesis 1; Hypothesis 2). The patients’ perceived value of insurance coverage was positively related to both satisfaction and trust (*P* < 0.001) (Hypothesis 3; Hypothesis 4). In addition, patients’ satisfaction with insurance was negatively related to their complaints about insurance and positively related to their trust in their insurance programs as hypothesized (*P* < 0.001) (Hypothesis 5; Hypothesis 6). The fulfillment of patients’ expectations of insurance benefits had the largest effect on their satisfaction with health insurance (coefficient = 0.593, *P* < 0.001), while the patients’ perceived value of insurance coverage had the largest impact on their trust in health insurance (coefficient = 0.409, *P* < 0.01).

The exogenous variables (awareness of insurance policies, fulfillment of expectations of insurance benefits, and perceived value of insurance coverage) had indirect effects on patients’ complaints and trust (Table 2). These variables also exerted direct effects on patients’ satisfaction with insurance. Thus, it could be inferred that patients’ satisfaction with insurance played a mediating role in the hypothesized model, and Hypothesis 7 was proved.

**Table 2:** Standardized direct and indirect effects of the hypothesized model (*n* = 922)

Type of effects	Path	Mean effects	S.E.
Direct effects	Policy awareness ➞ Satisfaction	0.183^**^	0.028
	Fulfillment of expectations ➞ Satisfaction	0.593^**^	0.027
	Perceived value ➞ Satisfaction	0.165^**^	0.030
	Perceived value ➞ Trust	0.409^*^	0.045
	Satisfaction ➞ Trust	0.236^**^	0.041
	Satisfaction ➞ Complaints	−0.443^**^	0.038
Indirect efects	Policy awareness ➞ Complaints	−0.081^**^	0.014
	Fulfillment of expectations ➞ Complaints	−0.263^**^	0.025
	Perceived value ➞ Complaints	−0.073^**^	0.015
	Policy awareness ➞ Trust	0.043^**^	0.009
	Fulfillment of expectations ➞ Trust	0.140^**^	0.026
	Perceived value ➞ Trust	0.039^**^	0.010

*S.E.* standard error; ^*^P < 0.01; ^**^P < 0.001

### Multi-group analysis by type of insurance

Compared to URRBMI patients, UEBMI patients had a higher degree of satisfaction with insurance on average, in terms of the scope of healthcare services covered, reimbursement ratio, reimbursement amount, the efficiency of reimbursement procedure, and overall reimbursement experience (Table 3). Although differences in the degree of satisfaction by type of insurance, the hypothesized paths were statistically significant in UEBMI and URRBMI groups, and main findings from the SEM were also proved by the multi-group analysis (Table 4).

**Table 3:** One-way ANOVA of the variations in satisfaction by type of insurance

Variables	UEBMI (*n* = 516)		URRBMI (*n* = 406)		T-statistics
	Mean	S.D.	Mean	S.D.	
The scope of healthcare services covered by insurance program	4.34	1.325	4.03	1.224	3.716^**^
The proportion of medical expenditure reimbursed by insurance program	4.38	1.316	3.99	1.251	4.634^**^
The reimbursement amount by insurance program for outpatient services	4.18	1.341	3.86	1.260	3.701^**^
The reimbursement amount by insurance program for inpatient services	4.41	1.312	4.01	1.263	4.638^**^
The efficiency of reimbursement procedure	4.49	1.261	4.22	1.234	3.259^*^
The overall insurance reimbursement experience	4.55	1.226	4.19	1.140	4.639^**^

*S.D.* standard deviation; ^*^P < 0.01; ^**^P < 0.001

**Table 4:** Standardized path coefficients of the multi-group analysis

Path	UEBMI (n = 516)		URRBMI (n = 406)	
	Mean effects	S.E.	Mean effects	S.E.
Policy awareness ➞ Satisfaction	0.207^**^	0.036	0.130^**^	0.048
Fulfillment of expectations ➞ Satisfaction	0.650^**^	0.031	0.506^**^	0.049
Perceived value ➞ Satisfaction	0.132^**^	0.038	0.217^**^	0.048
Perceived value ➞ Trust	0.400^**^	0.060	0.412^**^	0.067
Satisfaction ➞ Trust	0.264^**^	0.055	0.181^**^	0.058
Satisfaction ➞ Complaints	−0.477^**^	0.049	−0.370^**^	0.063

*S.E.* standard error; ^**^P < 0.001

Despite the equality constraints imposed on both factor loadings and regression weights, the model of the structured weight fitted the data well (e.g., CFI = 0.967) and demonstrated an acceptable approximation (RMSEA = 0.039) (Additional file 1: Table S6). The constrained and unconstrained models did not significantly differ in measurement weights (*P* = 0.053) and structural covariances (*P* = 0.191) (Additional file 1: Table S7). Although there was statistical significance (*P* = 0.019) in the structural weights, the CFI difference test met the cut-off criteria of less than 0.01. As Cheung and Rensvold [63] reasoned that it could be more rational to base invariance decisions on a difference in CFI (ΔCFI) rather than on χ^2^ values, as the χ^2^ difference test was an excessively stringent test of invariance [64, 65]. Thus, the constructs of the model were reasonably invariant and thus comparable across UEBMI and URRBMI groups.

## Discussion

Owing to the rising burden of chronic diseases, healthcare systems in many countries are struggling to keep pace with the increased health expectations and healthcare demand by patients with chronic diseases, especially as the countries are making progress towards UHC [66]. Our study provided information about satisfaction with health insurance by patients with chronic diseases after China had established its UHC. We proposed a hypothesized model and provided empirical evidence on patients’ satisfaction with insurance programs. Our results showed that patients’ awareness of health insurance policies, their insurance programs’ fulfillment of their expectations of insurance benefits, and their perceived value of insurance coverage positively predicted their satisfaction with insurance. Among the exogenous variables, the fulfillment of patients’ expectations of insurance benefits had the largest effect on satisfaction with insurance. Patients’ perceived value of insurance coverage not only affected their satisfaction with insurance but also had a direct impact on the degree of trust in insurance. Moreover, our findings suggested that patients with a high level of satisfaction with insurance had a higher degree of trust in the insurance and few complaints about insurance.

One SEM study with 483 valid questionnaires from the NRCMS enrollees found that patients’ perceived reliability of health insurance had a significant positive effect on satisfaction but did not have a significant effect on continued insurance enrollment [22]. In that study, trust was part of the manifest variables related to the perceived reliability, compared with trust as an independent latent variable in our research. Chen W et al. conducted a cross-sectional study to evaluate the effects of trust in primary care physicians on satisfaction with general medical services among hypertension patients in rural China [67]. They concluded that patients’ trust in physicians’ benevolence had a positive effect on their satisfaction [67]. However, our study had different research objectives and distinct hypotheses. We found that a higher level of satisfaction with insurance by patients increased the trust in insurance programs. Recent studies [68–70] also proved that increase in patient satisfaction could have a positive impact on patients’ trust. Another SEM research on URRBMI enrollees suggested that the perception of policy quality was a decisive factor in the perceived quality of URRBMI [20]. Given our results that patients’ awareness of their insurance policies was very limited, it seemed to be a little bit difficult for patients to make a non-biased assessment of the policy quality.

Although different variables, participants, and models were used by prior health insurance studies [20, 22] as compared with our analysis, similar conclusions were drawn in terms of key factors influencing patient satisfaction, such as reimbursement ratio, the scope of healthcare services, and technologies listed in the insurance catalog. On the other hand, we identified several new findings that had not been reported by prior research.

First, we found a low level of awareness of the insurance policies among the study patients. Our finding suggests that policymakers need to increase patients’ awareness of their health insurance policies so that they can understand and take full advantage of insurance benefits, and subsequently have higher levels of satisfaction with and trust in insurance programs. Specifically, the NHSA and its branches need to formulate and implement communication strategies by using various methods, such as conventional brochures and social media [71], to increase patients’ knowledge and understanding of insurance benefits and the policies and procedures of getting reimbursed for medical expenditure [72].

Second, the major predictor for patient satisfaction with health insurance was the fulfillment of patient expectations of insurance benefits. Our results can be helpful for policymakers to focus on patient-centered care and patient satisfaction by optimizing insurance schemes to meet patients’ expectations. Policymakers are suggested to pay close attention to patients’ demands and rationally formulate evidence-informed reimbursement policies that meet patients’ expectations. The significant effect of expectation fulfillment on patient satisfaction, underscores the importance of health insurance performance, as their expectations strongly depend on factors such as the reimbursed healthcare service, the copayment ratio, and the compensation amount.

Third, patients’ perceived value of insurance coverage had direct and significant positive effects on their trust in health insurance. Our finding highlights the importance of incorporating patient perspectives in making health insurance policies [73]. Even though it has been rare to engage patients in China’s health insurance decision-making process, our finding suggests that policymakers in China should at least measure patients’ perspective of health insurance as part of their ongoing efforts to increase satisfaction with health insurance by patients, especially those who have chronic diseases.

Finally, we found a higher degree of satisfaction by UEBMI patients, compared with URRBMI patients. One possible explanation for the finding is the disparities in insurance benefits and healthcare utilization between the two groups. As previous studies showed, URRBMI patients had a significantly shorter length of hospital stay and higher out-of-pocket expenditures when compared with concurrent UEBMI patients [74–76]. To increase satisfaction with health insurance among URRBMI patients, China’s policymakers need to make further efforts to improve URRBMI benefits, especially its reimbursement level.

Our research applied a patient-centered approach to reflect patient’s views of and satisfaction with health insurance. The SEM allowed us to test several relationships simultaneously within a conceptual model, which is important for patient satisfaction research where many mediating variables are suspected to have complex inter-correlations. Unlike other studies [20, 22], the participants in our SEM were patients with chronic diseases who had actual experiences of getting reimbursed for their outpatient and/or inpatient care. Consequently, their views can truly reflect the real-world performance of insurance programs. Motivated by the inequalities in reimbursement rates and service coverage between UEBMI and URRBMI, we conducted a multi-group SEM analysis by type of insurance, which was seldom used by previous studies of patient satisfaction with health insurance in China.

Notwithstanding the strengths, there are several limitations in our study. First, our survey was performed in tertiary hospitals, where patients were likely to have more severe diseases and higher demand for healthcare services than the patients in secondary and primary hospitals. Second, our sampled hospitals were located in Jiangsu province, one of the most economically developed regions in China. Future studies are warranted to have nationally representative samples. Finally, our research provides suggestions under current health insurance policies in China. Future studies need to develop more accurate measurement variables and survey items according to different health insurance policy settings and perform comprehensive assessments on patient satisfaction.

## Conclusions

Our study established a hypothesized model on satisfaction with China’s public health insurance programs by patients with chronic diseases. Our empirical analysis confirmed significant effects on patients’ satisfaction of key factors (e.g., patients’ awareness of health insurance policies, insurance programs’ fulfillment of their expectations of insurance benefits, and their perceived value of insurance coverage). Our findings have important implications for policymakers who are taking initiatives to sustain the country’s UHC.

## Supplementary Information

**Additional file 1.**

## Data Availability

Original datasets will be available upon reasonable request to the corresponding author.

## Abbreviations

UHC: Universal health insurance coverage
UEBMI: Urban Employee Basic Medical Insurance
NRCMS: New Rural Cooperative Medical Scheme
URBMI: Urban Resident Basic Medical Insurance
URRBMI: Urban-Rural Resident Basic Medical Insurance
SEM: Structural equation modeling
NHSA: National Healthcare Security Administration
CR: Composite reliability
AVE: Average variance extracted
CFI: Comparative fit index
RMSEA: Root mean square error of approximation

## References

[1] Lu J, Lu Y, Wang X, Li X, Linderman GC, Wu C, Cheng X, Mu L, Zhang H, Liu J, Su M, Zhao H, Spatz ES, Spertus JA, Masoudi FA, Krumholz HM, Jiang L (2017). Prevalence, awareness, treatment, and control of hypertension in China: data from 1.7 million adults in a population-based screening study (China PEACE million persons project). Lancet..

[2] Williams R, Karuranga S, Malanda B, Saeedi P, Basit A, Besançon S (2020). Global and regional estimates and projections of diabetes-related health expenditure: results from the International Diabetes Federation Diabetes Atlas, 9th edition. Diabetes Res Clin Pract.

[3] Bloom DE, Chen S, Kuhn M, McGovern ME, Oxley L, Prettner K. The economic burden of chronic diseases: estimates and projections for China, Japan, and South Korea. JEoA. 2018;17:100163. 10.1016/j.jeoa.2018.09.002.

[4] Yu H (2015). Universal health insurance coverage for 1.3 billion people: what accounts for China's success?. Health Policy..

[5] Meng Q, Mills A, Wang L, Han Q (2019). What can we learn from China's health system reform?. BMJ..

[6] Xian W, Xu X, Li J, Sun J, Fu H, Wu S, Liu H (2019). Health care inequality under different medical insurance schemes in a socioeconomically underdeveloped region of China: a propensity score matching analysis. BMC Public Health.

[7] Zhao M, Liu B, Shan L, Li C, Wu Q, Hao Y, Chen Z, Lan L, Kang Z, Liang L, Ning N, Jiao M (2019). Can integration reduce inequity in healthcare utilization? Evidence and hurdles in China. BMC Health Serv Res.

[8] Yip W, Fu H, Chen AT, Zhai T, Jian W, Xu R, Pan J, Hu M, Zhou Z, Chen Q, Mao W, Sun Q, Chen W (2019). 10 years of health-care reform in China: progress and gaps in universal health coverage. Lancet..

[9] Shan L, Zhao M, Ning N, Hao Y, Li Y, Liang L, Kang Z, Sun H, Ding D, Liu B, Liang C, Yu M, Wu Q, Hao M, Fan H (2018). Dissatisfaction with current integration reforms of health insurance schemes in China: are they a success and what matters?. Health Policy Plan.

[10] World Health Organization (2016). Annex 2: Illustrations of the application of mixing action domains for addressing commonly faced challenges in the region. World Health Organization. Universal health coverage: moving towards better health: action framework for the Western Pacific Region.

[11] China’s National Healthcare Security Administration (2020). Guiding opinions of the National Healthcare Security Administration, the Ministry of Finance and the State Taxation Administration on strengthening and improving the particiaption in basic medical insurance. Policies and regulations.

[12] Arntsen B, Torjesen DO, Karlsen TI (2020). Associations between structures, processes and outcomes in inter-municipal cooperation in out-of-hours services in Norway: a survey study. Soc Sci Med.

[13] Cameron JE, Voth J, Jaglal SB, Guilcher SJT, Hawker G, Salbach NM (2018). "In this together": social identification predicts health outcomes (via self-efficacy) in a chronic disease self-management program. Soc Sci Med.

[14] Weinhold I, Gurtner S (2018). Rural - urban differences in determinants of patient satisfaction with primary care. Soc Sci Med.

[15] Sommers BD, Gawande AA, Baicker K (2017). Health insurance coverage and health - what the recent evidence tells us. N Engl J Med.

[16] Zhang M, Wang H (2015). Application of structural equation model in the analysis of satisfaction with basic social medical insurance in Shanxi Province. Chin J Health Stat.

[17] Yu B, Jin T, Shen J, Wu H (2019). Research on satisfaction and influencing factors of basic medical insurance for urban and rural resident: taking Shanghai as an example. Price: Theory & Practice.

[18] Wang Q, Mo X, Peng L, Shi J, Lei S, Lin Q (2015). Evaluation of a satisfaction index model for rural and urban pupils with basic medical insurance. Chin J Health Stat..

[19] Yang H, Yang Y (2017). Study on the satisfaction of medical insurance scheme for urban and rural residents: illustrated by Ezhou City of Hubei Province. J Central Univ Financ Econ.

[20] Liu X, Yang F, Cheng W, Wu Y, Cheng J, Sun W, Yan X, Luo M, Mo X, Hu M, Lin Q, Shi J (2020). Mixed methods research on satisfaction with basic medical insurance for urban and rural residents in China. BMC Public Health.

[21] Liu X, Cheng W, Yan X, Peng L, Song X, Jiao F, Shi J, Xiao X (2020). Application of satisfaction index of basic medical insurance for rural and urban residents to pupils’ familial decision making in Kunming and Changsha City. J Central South Univ Med Sci.

[22] Gu D, Yang X, Li X, Liang C, Zhong J, Feng N (2018). Innovating new rural cooperative medical scheme (NCMS) for better patient satisfaction in rural China. Int J Environ Res Public Health.

[23] Tang L (2011). The influences of patient's trust in medical service and attitude towards health policy on patient's overall satisfaction with medical service and sub satisfaction in China. BMC Public Health.

[24] Cui C, Zuo X, Wang Y, Song H, Shi J, Meng K (2020). A comparative study of patients' satisfaction with different levels of hospitals in Beijing: why do patients prefer high-level hospitals?. BMC Health Serv Res.

[25] Mohammed S, Sambo MN, Dong H (2011). Understanding client satisfaction with a health insurance scheme in Nigeria: factors and enrollees experiences. Health Res Policy Syst.

[26] Becker JA (2013). Examining relationships between hospital inpatient expectations and satisfaction for maximum Medicare reimbursement.

[27] Jain D, Bendich I, Nguyen LL, Nguyen LL, Lewis CG, Huddleston JI (2017). Do patient expectations influence patient-reported outcomes and satisfaction in total hip arthroplasty? A prospective, multicenter study. J Arthroplast.

[28] Neuprez A, Delcour JP, Fatemi F, Gillet P, Crielaard JM, Bruyère O, Reginster JY (2016). Patients' expectations impact their satisfaction following total hip or knee arthroplasty. PLoS One.

[29] Gonzalez Sáenz de Tejada M, Escobar A, Herrera C, García L, Aizpuru F, Sarasqueta C (2010). Patient expectations and health-related quality of life outcomes following total joint replacement. Value Health.

[30] Deakin AH, Smith MA, Wallace DT, Smith EJ, Sarungi M (2019). Fulfilment of preoperative expectations and postoperative patient satisfaction after total knee replacement. A prospective analysis of 200 patients. Knee..

[31] Lee YC, Wang YC, Lu SC, Hsieh YF, Chien CH, Tsai SB, Dong W (2016). An empirical research on customer satisfaction study: a consideration of different levels of performance. SpringerPlus..

[32] Gera R, Mittal S, Batra DK, Prasad B (2017). Evaluating the effects of service quality, customer satisfaction, and service value on behavioral intentions with life insurance customers in India. IJSSMET..

[33] Aljaberi MA, Juni MH, Al-Maqtari RA, Lye MS, Saeed MA, Al-Dubai SAR (2018). Relationships among perceived quality of healthcare services, satisfaction and behavioural intentions of international students in Kuala Lumpur, Malaysia: a cross-sectional study. BMJ Open.

[34] Abdelfattah FA, Rahman MS, Osman M (2015). Assessing the antecedents of customer loyalty on healthcare insurance products: service quality; perceived value embedded model. J Ind Eng Manag.

[35] Yilmaz V, Ari E (2017). The effects of service quality, image, and customer satisfaction on customer complaints and loyalty in high-speed rail service in Turkey: a proposal of the structural equation model. Transport..

[36] Stelfox HT, Gandhi TK, Orav EJ, Gustafson ML (2005). The relation of patient satisfaction with complaints against physicians and malpractice lawsuits. Am J Med.

[37] Lestariningsih T, Hadiyati E, Astuti R (2018). Study of service quality and patient satisfaction to trust and loyalty in public hospital. Indonesia IJBMM.

[38] Jing L, Chen R, Jing L, Qiao Y, Lou J, Xu J, Wang J, Chen W, Sun X (2017). Development and enrollee satisfaction with basic medical insurance in China: a systematic review and stratified cluster sampling survey. Int J Health Plann Manag.

[39] Xiong X, Zhang Z, Ren J, Zhang J, Pan X, Zhang L, Gong S, Jin S (2018). Impact of universal medical insurance system on the accessibility of medical service supply and affordability of patients in China. PLoS One.

[40] Xu J, Jian W, Zhu K, Kwon S, Fang H (2019). Reforming public hospital financing in China: progress and challenges. BMJ..

[41] World Health Organization (2010). Key components of a well functioning health system.

[42] Nadi A, Shojaee J, Abedi G, Siamian H, Abedini E, Rostami F (2016). Patients' expectations and perceptions of service quality in the selected hospitals. Med Arch.

[43] Bjertnaes OA, Sjetne IS, Iversen HH (2012). Overall patient satisfaction with hospitals: effects of patient-reported experiences and fulfilment of expectations. BMJ Qual Saf.

[44] Miao Y, Yuan X, Gu J, Zhang L, He R, Sandeep S, Wu J (2019). Constructing a value-based healthcare system for hypertensive patients through changing payment mode: evidence from a comparative study in rural China. J Med Econ.

[45] Wu Y, Zhang L, Liu X, Ye T, Wang Y (2018). Geographic variation in health insurance benefits in Qianjiang District, China: a cross-sectional study. Int J Equity Health.

[46] Pevec T, Pisnik A (2018). Empirical evaluation of a conceptual model for the perceived value of health services. Zdr Varst.

[47] Pan FFC (2011). Perceived values on hospital services: a fuzzy logic application. Afr J Bus Manage.

[48] Centers for Disease Control and Prevention (2015). V-BID approaches. Understanding value-based insurance design. National Center for Chronic Diease Prevention and Health Promotion.

[49] Turk D, Boeri M, Abraham L, Atkinson J, Bushmakin AG, Cappelleri JC, Hauber B, Klein K, Russo L, Viktrup L, Walsh D (2020). Patient preferences for osteoarthritis pain and chronic low back pain treatments in the United States: a discrete-choice experiment. Osteoarthr Cartil.

[50] Mansfield C, Gebben DJ, Sutphin J, Tepper SJ, Schwedt TJ, Sapra S, Shah N (2019). Patient preferences for preventive migraine treatments: a discrete-choice experiment. Headache..

[51] Wong XY, Lim AQJ, Shen Q, Chia JWK, Chew MH, Tan WS, Wee HL (2020). Patient preferences and predicted relative uptake for targeted therapies in metastatic colorectal cancer: a discrete choice experiment. Curr Med Res Opin.

[52] Wang XR, Wang HM (2016). Factor analysis of satisfaction on basic social medical insurances in Liaoning based on survey from 2011 to 2015. Chin Health Ser Manage.

[53] Harrison R, Walton M, Healy J, Smith-Merry J, Hobbs C (2016). Patient complaints about hospital services: applying a complaint taxonomy to analyse and respond to complaints. Int J Qual Health Care.

[54] Pichert JW, Hickson G, Moore I, Henriksen K, Battles JB, Keyes MA, Grady ML (2008). Advances in patient safety using patient complaints to promote patient safety. Advances in patient safety: new directions and alternative approaches (Vol 2: culture and redesign).

[55] Reader TW, Gillespie A, Roberts J (2014). Patient complaints in healthcare systems: a systematic review and coding taxonomy. BMJ Qual Saf.

[56] Lofgren C, Thanh NX, Chuc NT, Emmelin A, Lindholm L (2008). People’s willingness to pay for health insurance in rural Vietnam. Cost Eff Resour Alloc.

[57] Zhang L, Wang H, Wang L, Hsiao W (2006). Social capital and farmer's willingness-to-join a newly established community-based health insurance in rural China. Health Policy.

[58] Mitchell R, Scheiner EM, Gurevitch J (1993). Path analysis: pollination. Design and analysis of ecological experiments.

[59] Whittaker TA, Pituch KA, Stevens JP (2016). Structural equation modeling. Applied multivariate statistics for the social sciences. Analyses with SAS and IBM’s SPSS.

[60] Fornell C, Larcker DF (1981). Evaluating structural equation models with unobservable variables and measurement error. JMR..

[61] McDonald RP, Ho MHR (2002). Principles and practice in reporting structural equation analyses. Psychol Methods.

[62] Bollen KA, Bollen KA (1989). Measurement models: the relation between latent and observed variables. Structural equations with latent variables.

[63] Cheung GW, Rensvold RB (2002). Evaluating goodness-of-fit indexes for testing measurement invariance. Struct Equ Modeling.

[64] Cudeck R, Browne MW (1983). Cross-validation of covariance structures. Multivariate Behav Res.

[65] MacCallum RC, Roznowski M, Necowitz LB (1992). Model modifications in covariance structure analysis: the problem of capitalization on chance. Psychol Bull.

[66] Collaborators GUHC (2020). Measuring universal health coverage based on an index of effective coverage of health services in 204 countries and territories, 1990-2019: a systematic analysis for the global burden of disease study 2019. Lancet..

[67] Chen W, Feng Y, Fang J, Wu J, Huang X, Wang X, Wu J, Zhang M (2020). Effect of trust in primary care physicians on patient satisfaction: a cross-sectional study among patients with hypertension in rural China. BMC Fam Pract.

[68] Durmuş A, Akbolat M (2020). The impact of patient satisfaction on patient commitment and the mediating role of patient trust. J Patient Exp.

[69] Liu S, Li G, Liu N, Hongwei W (2021). The impact of patient satisfaction on patient loyalty with the mediating effect of patient trust. INQUIRY..

[70] Cahyati P (2021). The model of patient satisfaction and trust: a study at BPJS patient. DIJEMSS..

[71] Puljak L (2016). Using social media for knowledge translation, promotion of evidence-based medicine and high-quality information on health. JEBM..

[72] Tipirneni R, Politi MC, Kullgren JT, Kieffer EC, Goold SD, Scherer AM (2018). Association between health insurance literacy and avoidance of health care services owing to cost. JAMA Netw Open.

[73] Huang R, Gionfriddo MR, Zhang L, Leppin AL, Ting HH, Montori VM (2015). Shared decision-making in the People’s republic of China: current status and future directions. Patient Prefer Adherence.

[74] Chen H, Shi L, Xue M, Wang N, Dong X, Cai Y, Chen J, Zhu W, Xu H, Meng Q (2018). Geographic variations in in-hospital mortality and use of percutaneous coronary intervention following acute myocardial infarction in China: a nationwide cross-sectional analysis. J Am Heart Assoc.

[75] Lin X, Cai M, Tao H, Liu E, Cheng Z, Xu C, Wang M, Xia S, Jiang T (2017). Insurance status, inhospital mortality and length of stay in hospitalised patients in Shanxi, China: a cross-sectional study. BMJ Open.

[76] Bai L, Wushouer H, Huang C, Luo Z, Guan X, Shi L (2020). Health care utilization and costs of patients with prostate cancer in China based on national health insurance database from 2015 to 2017. Fron Pharmacol.

