# Supplementary material for: Assessment of the satisfaction with public health insurance programs by patients with chronic diseases in China: a structural equation modeling approach
Source: BMC Public Health. 2021 Oct 19;21:1886. doi: 10.1186/s12889-021-11947-7 (PMC8524814; doi:10.1186/s12889-021-11947-7)
Supplement: Supplementary file 1 — Additional file 1. [file 12889_2021_11947_MOESM1_ESM.pdf]

Appendix

Table S1. Measurements of the latent variables

Latent variables	Manifest variables	Survey items
Patients' awareness of insurance policies	X1	I am familiar with the catalog of the health technologies that are covered by my insurance program and relevant to my chronic disease.
	X2	I am familiar with the deductibles required by my insurance program.
	X3	I am familiar with the cap of my insurance reimbursement.
	X4	I am familiar with the copayment ratio.
	X5	I am familiar with the reimbursement procedure.
Fulfillment of patients' expectations of insurance benefits	X6	The scope of reimbursed healthcare services fulfills my pre-reimbursement expectations.
	X7	The proportion of medical expenditure covered by my insurance program meets my pre-reimbursement expectations.
	X8	The total amount of reimbursed medical expenditure meets my pre-reimbursement expectations.
Patients' perceived value of insurance coverage	X9	The clinical effectiveness of the covered medical technologies is good.
	X10	The insurance is helpful to reduce my financial burden of medical expenditure.
	X11	Due to the insurance, I need less support from my family members and friends.
Patients' satisfaction with insurance		How satisfied are you with:
	Y1	The scope of healthcare services covered by my insurance program.
	Y2	The proportion of medical expenditure reimbursed by my insurance program.
	Y3	The reimbursement amount by my insurance program for outpatient services.
	Y4	The reimbursement amount by my insurance program for inpatient services.
	Y5	The efficiency of reimbursement procedure.
	Y6	The overall insurance reimbursement experience.
Patients' complaints about insurance	Y7	I have many complaints about my health insurance program.
	Y8	I often try to find solutions to my complaints by calling or writing to the NHSA and its branches or other agencies.
Patients' trust in insurance	Y9	The medical technologies covered by my insurance program were worth using.
	Y10	I am willing to remain enrolled in the health insurance scheme.
	Y11	I believe that the health insurance scheme will keep improving.

Table S2. Number of participants in the sampled hospitals

Name of hospital	City	Number of patients
Affiliated Hospital of Nantong University	Nantong	296
Nantong First People's Hospital	Nantong	31
Taizhou People's Hospital	Taizhou	134
Yancheng No.1 People's Hospital	Yancheng	155
The First Affiliated Hospital of Soochow University	Suzhou	60
Changshu No.1 People's Hospital	Suzhou	60
Suzhou Ninth People's Hospital	Suzhou	21
Wuxi No.2 People's Hospital	Wuxi	87
Wuxi No.3 People's Hospital	Wuxi	53
Jiangyin People's Hospital	Wuxi	25

Table S3. Reliability and validity of measurement scales and item-construct loadings

Latent variables	Cronbach's α	CR	AVE	Manifest variables	Factor loadings	Mean (SD)
Patients' awareness of insurance policies	0.903	0.893	0.625	X1	0.827	3.385 (1.492)
				X2	0.733	3.256 (1.560)
				X3	0.750	3.079 (1.457)
				X4	0.847	3.744 (1.554)
				X5	0.790	3.860 (1.530)
Fulfillment of patients' expectations of insurance benefits	0.941	0.941	0.843	X6	0.884	4.073 (1.364)
				X7	0.947	3.992 (1.347)
				X8	0.922	4.007 (1.356)
Patients' perceived value of insurance coverage	0.939	0.939	0.838	X9	0.889	5.547 (1.176)
				X10	0.945	5.612 (1.176)
				X11	0.911	5.700 (1.146)
Patients' satisfaction with insurance	0.941	0.939	0.723	Y1	0.894	4.207 (1.290)
				Y2	0.922	4.210 (1.302)
				Y3	0.865	4.039 (1.315)
				Y4	0.934	4.232 (1.305)
				Y5	0.603	4.375 (1.256)
				Y6	0.840	4.393 (1.202)
Patients' complaints about insurance	0.912	0.913	0.841	Y7	0.941	3.222 (1.422)
				Y8	0.892	3.017 (1.390)
Patients' trust in insurance	0.826	0.826	0.614	Y9	0.740	5.194 (1.297)
				Y10	0.861	5.680 (1.157)
				Y11	0.744	5.492 (1.202)

Note: Cronbach's α should exceed 0.7; Composite reliability (CR) should be larger than 0.7; Average variance extracted (AVE) should be greater than 0.5; Standard deviation (SD)

Table S4. Discriminant validity of the latent variables

Latent variables	Policy awareness	Fulfillment of expectations	Perceived value	Satisfaction	Complaints	Trust
Policy awareness	**0.791**	0.026	0.013	0.088	0.017	0.014
Fulfillment of expectations	0.160	**0.918**	0.105	0.457	0.089	0.085
Perceived value	0.114	0.324	**0.915**	0.143	0.028	0.248
Satisfaction	0.296	0.676	0.378	**0.850**	0.196	0.152
Complaints	-0.131	-0.299	-0.167	-0.443	**0.917**	0.030
Trust	0.117	0.292	0.498	0.390	-0.173	**0.784**

On the diagonal are the square-root value of the AVE; below the diagonal are the correlation values; and above the diagonal are the squares of the correlation values.

Table S5. Goodness-of-fit results of the hypothesized model

Statistics		Values	Desired range of values for good fit
Absolute fit indices			
Standardized root mean square residual	SRMR	0.046	<0.08
Root mean square error of approximation	RMSEA	0.049	<0.08
Goodness-of-fit index	GFI	0.940	>0.90
Adjusted goodness-of-fit index	AGFI	0.923	>0.90
Chi-square/degrees of freedom ratio	χ^2/df	3.207	2～5
Hoelter's Critical N	CN	359	>200
Incremental fit indices			
Tucker-Lewis index	TLI	0.971	>0.90
Normed fit index	NFI	0.964	>0.90
Comparative fit index	CFI	0.975	>0.90
Relative fit index	RFI	0.958	>0.90
Incremental fit index	IFI	0.975	>0.90
Parsimonious fit indices			
Parsimony comparative fit index	PCFI	0.832	>0.50
Parsimony normed fit index	PNFI	0.822	>0.50
Parsimony goodness-of-fit index	PGFI	0.732	>0.50

Table S6. Goodness-of-fit results of the multi-group analysis

Statistics		Unconstrained model	Measurement weights model	Structural weights model	Structural covariances model
Absolute fit indices					
Standardized root mean square residual	SRMR	0.055	0.057	0.061	0.064
Root mean square error of approximation	RMSEA	0.039	0.039	0.039	0.039
Goodness-of-fit index	GFI	0.913	0.911	0.910	0.908
Adjusted goodness-of-fit index	AGFI	0.888	0.890	0.891	0.890
Chi-square/degrees of freedom ratio	χ^2/df	2.428	2.373	2.369	2.371
Hoelter's Critical N	CN	446	455	455	454
Incremental fit indices					
Tucker-Lewis index	TLI	0.962	0.963	0.963	0.963
Normed fit index	NFI	0.946	0.945	0.944	0.944
Comparative fit index	CFI	0.967	0.967	0.967	0.967
Relative fit index	RFI	0.937	0.938	0.938	0.938
Incremental fit index	IFI	0.968	0.968	0.967	0.967
Parsimonious fit indices					
Parsimony comparative fit index	PCFI	0.825	0.859	0.871	0.883
Parsimony normed fit index	PNFI	0.807	0.839	0.850	0.862
Parsimony goodness-of-fit index	PGFI	0.711	0.738	0.748	0.758

Table S7. Noninvariance test of the multi-group analysis

Statistics		Measurement weights model	Structural weights model	Structural covariances model
ΔChi-square	$\Delta\chi^2$	12.452	15.112	4.748
ΔDegrees of freedom	ΔDF	6	6	3
Probability according to ΔChi-square and ΔDegrees of freedom	P	0.053	0.019	0.191
ΔTucker-Lewis index	ΔTLI	0.000	0.000	0.000
ΔNormed fit index	ΔNFI	0.001	0.001	0.000
ΔComparative fit index	ΔCFI	0.000	0.000	0.000
ΔRelative fit index	ΔRFI	0.000	0.000	0.000
ΔIncremental fit index	ΔIFI	0.001	0.001	0.000

Δ refers to each of the increasingly more restrictive models from the unconstrained to the structural covariances
